# [2,5-Bis(di­propyl­amino)-4-(hy­droxy­meth­yl)phen­yl]methanol

**DOI:** 10.1107/S2414314621004430

**Published:** 2021-04-30

**Authors:** Volker Schmitt, Gideon Holzmann, Dieter Schollmeyer, Heiner Detert

**Affiliations:** a University of Mainz, Institut for Organic Chemistry, Duesbergweg 10-14, 55099 Mainz, Germany; University of Aberdeen, Scotland

**Keywords:** crystal structure, phenyl­enedi­amine, alcohol, hydrogen bond

## Abstract

The centrosymmetric title compound, C_22_H_36_N_2_O_2_, was prepared in five steps from diethyl succinate. The di­propyl­amino groups are almost orthogonal to the central phenyl­enedi­methanol ring [dihedral angle = 87.62 (9)°]. In the crystal, the mol­ecules are connected by O—H⋯N hydrogen bonds, forming (101) layers separated by the propyl chains.

## Abstract 2

The centrosymmetric title compound was prepared in five steps from diethyl succinate. The di­propyl­amino groups are almost orthogonal to the central phenyl­enedi­methanol ring system [dihedral angle = 87.62 (9)°]. In the crystal, the mol­ecules are connected by O—H⋯N hydrogen bonds, forming (101) layers separated by the propyl chains.

## Graphical abstract

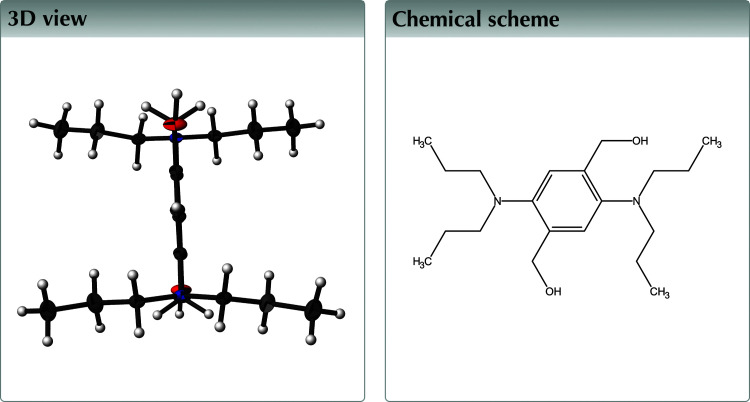

## Structure description

In a project focusing on acidochromic oligo­phenyl­ene­vinyl­enes (Detert *et al.*, 2004 ▸; Detert & Sugiono, 2004 ▸, 2005 ▸), the title compound, C_22_H_36_N_2_O_2_, was prepared as an inter­mediate for fluoro­phores with a central *p*-amino­aniline unit (Detert & Schmitt, 2004 ▸, 2006 ▸; Schmitt *et al.*, 2008 ▸).

**Figure 1:**
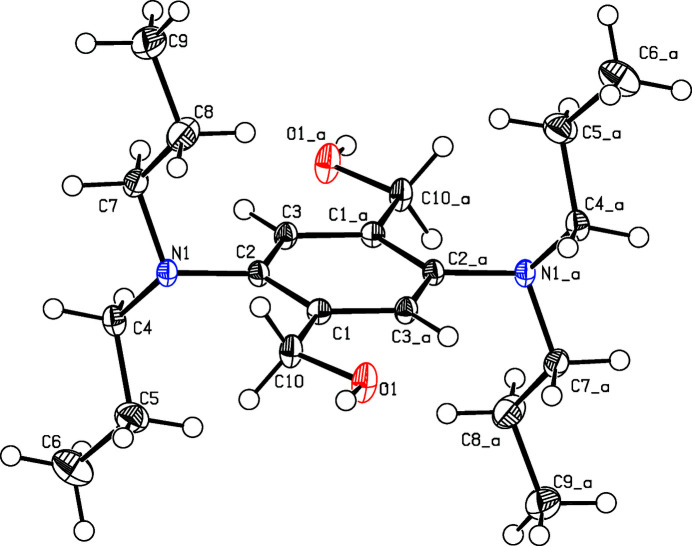
The mol­ecular structure with displacement ellipsoids drawn at the 50% probability level. Symmetry code: (a) 1 − *x*, 1 − *y*, −*z*.

The complete mol­ecule is generated by a crystallographic centre of symmetry (Fig. 1 ▸) and two centrosymmetric mol­ecules occupy the monoclinic unit cell. The mol­ecules are composed of an almost planar aromatic ring flanked by prolate di­propyl­amino groups. The mean planes of the ring and the di­propyl­amino unit enclose a dihedral angle of 87.62 (9)°. This orientation and torsion angles of −118.97 (11)° (C1—C2—N1—C4) and 0.8 (2)° (O1—C10—C1—C3_a) lead to an H-shape for the mol­ecule.

**Figure 2:**
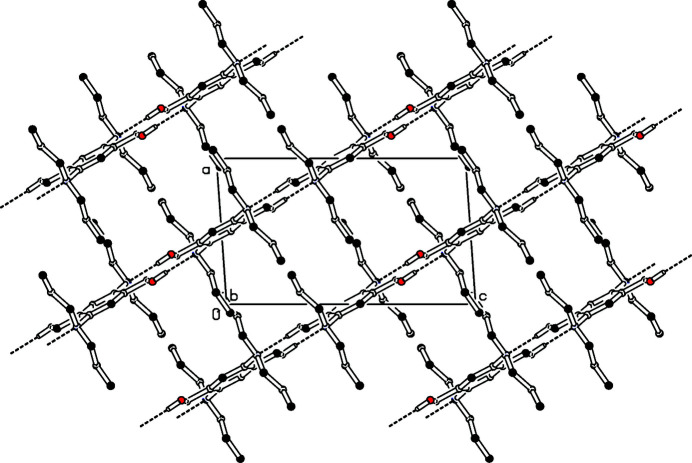
Partial packing diagram viewed along the *b*-axis direction. Hydrogen bonds are shown as dashed lines.

In the extended structure, slightly bent O—H⋯N hydrogen bonds (Table 1 ▸, Fig. 2 ▸) connect each mol­ecule with four neighbours, thus forming a slightly undulating network with an angle of 19.9° between the mean planes of the aromatic rings of adjacent mol­ecules. This network lies parallel to (101) and the propyl groups act as spacers between the planes.

## Synthesis and crystallization

The title compound was prepared from succinoyl succinate (Fehling, 1844 ▸) *via* condensation with propyl amine (Liebermann, 1914 ▸; Ulbricht *et al.*, 1979 ▸), refluxing of the di­amine with propionyl chloride for 3 h followed by aqueous work-up and recrystallization of the di­amide from toluene solution with *ca* 10% ethyl acetate. The amide (14 g, 0.033 mol) was added slowly to a stirred and boiling suspension of lithium aluminium hydride (3.8 g, 0.1 mol) in 200 ml of ether. After refluxing for 3 h, excess hydride was destroyed by addition of first ethyl acetate, and then aqueous sodium hydroxide (40%) to the stirred solution until clotting occurred. Suction filtration and digesting of the filter cake with ether, and washing of the combined organic phases with brine gave, after concentration and crystallization, 2.4 g (22%) of the di­amine. Recrystallization from aceto­nitrile solution resulted in 2.4 g (22%) of slightly yellowish cuboid crystals with m.p. = 400–401 K. ^1^H-NMR (400 MHz, CDCl_3_): 6.93 (*s*, 2 H ar); 4.75 (*s*, 4 H, benzylic), 2.83 (*m*, 8 H, N—CH2); 1.45 (*m*, 8 H), 0.85 (*t*, 12 H, CH_3_); ^13^C-NMR (100 MHz, CDCl_3_) 146.2 (C-2,5), 136.2 (C-1,4), 122.2 (C3,6), 64.8 (CH_2_OH), 57.0 (NCH_2_), 20.3 (CH_2_), 11.5 (CH_3_); FD—MS: *m*/*z* = 336.2 (100%, *M* ^+^); IR (CDCl_3_, cm^−1^): 3370, 2960, 2940, 2870, 1630, 1500, 1455, 1410, 1285, 1255, 1140, 1055.

## Refinement

Crystal data, data collection and structure refinement details are summarized in Table 2 ▸. Hydrogen atoms were located in difference Fourier maps and refined with isotropic displacement parameters.

## Supplementary Material

Crystal structure: contains datablock(s) I, global. DOI: 10.1107/S2414314621004430/hb4382sup1.cif

Structure factors: contains datablock(s) I. DOI: 10.1107/S2414314621004430/hb4382Isup2.hkl

Supporting information file. DOI: 10.1107/S2414314621004430/hb4382Isup3.cml

CCDC reference: 2080018

Additional supporting information:  crystallographic information; 3D view; checkCIF report

## Figures and Tables

**Table 1:** Hydrogen-bond geometry (Å, °)

*D*—H⋯*A*	*D*—H	H⋯*A*	*D*⋯*A*	*D*—H⋯*A*
O1—H1⋯N1^i^	0.88 (2)	2.05 (2)	2.9269 (13)	171.7 (18)

Symmetry code: (i) 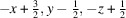 .

**Table 2:** Experimental details

Crystal data	
Chemical formula	C_20_H_36_N_2_O_2_
*M*_r_	336.51
Crystal system, space group	Monoclinic, *P*2_1_/*n*
Temperature (K)	120
*a*, *b*, *c* (Å)	8.2294 (4), 8.4573 (5), 14.0115 (7)
β (°)	93.974 (4)
*V* (Å^3^)	972.83 (9)
*Z*	2
Radiation type	Mo *K*α
μ (mm^−1^)	0.07
Crystal size (mm)	0.25 × 0.20 × 0.16

Data collection	
Diffractometer	Stoe IPDS 2T
Absorption correction	–
No. of measured, independent and observed [*I* > 2σ(*I*)] reflections	5278, 2318, 1995
*R*_int_	0.019
(sin θ/λ)_max_ (Å^−1^)	0.658

Refinement	
*R*[*F* ^2^ > 2σ(*F* ^2^)], *wR*(*F* ^2^), *S*	0.043, 0.112, 1.05
No. of reflections	2318
No. of parameters	172
H-atom treatment	All H-atom parameters refined
Δρ_max_, Δρ_min_ (e Å^−3^)	0.43, −0.16

Computer programs: *X-AREA* *WinXpose*, *Recipe* and *Integrate* (Stoe & Cie, 2019 ▸), *SHELXT2014* (Sheldrick, 2015*a* ▸), *SHELXL2018/3* (Sheldrick, 2015*b* ▸) and *PLATON* (Spek, 2020 ▸).

## full crystallographic data

### Crystal data

**Table:**

C_20_H_36_N_2_O_2_	*F*(000) = 372
*M**_r_* = 336.51	*D*_x_ = 1.149 Mg m^−^^3^
Monoclinic, *P*2_1_/*n*	Mo *K*α radiation, λ = 0.71073 Å
*a* = 8.2294 (4) Å	Cell parameters from 7030 reflections
*b* = 8.4573 (5) Å	θ = 2.4–28.4°
*c* = 14.0115 (7) Å	µ = 0.07 mm^−^^1^
β = 93.974 (4)°	*T* = 120 K
*V* = 972.83 (9) Å^3^	Block, colourless
*Z* = 2	0.25 × 0.20 × 0.16 mm

### Data collection

**Table:**

STOE IPDS 2T diffractometer	1995 reflections with *I* > 2σ(*I*)
Radiation source: sealed X-ray tube, 12 x 0.4 mm long-fine focus	*R*_int_ = 0.019
Detector resolution: 6.67 pixels mm^-1^	θ_max_ = 27.9°, θ_min_ = 2.8°
rotation method, ω scans	*h* = −9→10
5278 measured reflections	*k* = −11→11
2318 independent reflections	*l* = −18→18

### Refinement

**Table:**

Refinement on *F*^2^	Primary atom site location: dual
Least-squares matrix: full	Hydrogen site location: difference Fourier map
*R*[*F*^2^ > 2σ(*F*^2^)] = 0.043	All H-atom parameters refined
*wR*(*F*^2^) = 0.112	*w* = 1/[σ^2^(*F*_o_^2^) + (0.0491*P*)^2^ + 0.4938*P*] where *P* = (*F*_o_^2^ + 2*F*_c_^2^)/3
*S* = 1.05	(Δ/σ)_max_ < 0.001
2318 reflections	Δρ_max_ = 0.43 e Å^−^^3^
172 parameters	Δρ_min_ = −0.16 e Å^−^^3^
0 restraints	

### Special details

**Table:**

Geometry. All esds (except the esd in the dihedral angle between two l.s. planes) are estimated using the full covariance matrix. The cell esds are taken into account individually in the estimation of esds in distances, angles and torsion angles; correlations between esds in cell parameters are only used when they are defined by crystal symmetry. An approximate (isotropic) treatment of cell esds is used for estimating esds involving l.s. planes.

### Fractional atomic coordinates and isotropic or equivalent isotropic displacement parameters (Å^2^)

**Table:**

	*x*	*y*	*z*	*U*_iso_*/*U*_eq_	
O1	0.65711 (12)	0.27293 (10)	0.20873 (7)	0.0244 (2)	
H1	0.717 (3)	0.257 (2)	0.2627 (16)	0.046 (5)*	
N1	0.65180 (12)	0.75248 (11)	0.10757 (7)	0.0148 (2)	
C1	0.57794 (13)	0.47022 (13)	0.09097 (8)	0.0146 (2)	
C2	0.57352 (13)	0.62308 (13)	0.05321 (8)	0.0141 (2)	
C3	0.49620 (14)	0.65103 (13)	−0.03682 (8)	0.0151 (2)	
H3	0.4942 (17)	0.7560 (16)	−0.0625 (10)	0.014 (3)*	
C4	0.53162 (15)	0.87545 (14)	0.12965 (8)	0.0179 (2)	
H4A	0.4663 (18)	0.9151 (18)	0.0694 (10)	0.018 (2)*	
H4B	0.5946 (18)	0.9662 (18)	0.1557 (10)	0.018 (2)*	
C5	0.41498 (18)	0.81758 (17)	0.20128 (10)	0.0268 (3)	
H5A	0.478 (2)	0.779 (2)	0.2577 (13)	0.033 (3)*	
H5B	0.354 (2)	0.728 (2)	0.1733 (13)	0.033 (3)*	
C6	0.3000 (2)	0.9476 (2)	0.23025 (12)	0.0369 (4)	
H6A	0.227 (3)	0.908 (3)	0.2782 (15)	0.053 (3)*	
H6B	0.231 (3)	0.985 (2)	0.1760 (16)	0.053 (3)*	
H6C	0.365 (3)	1.039 (3)	0.2574 (15)	0.053 (3)*	
C7	0.78539 (15)	0.82069 (14)	0.05542 (8)	0.0186 (3)	
H7A	0.8328 (19)	0.9104 (19)	0.0947 (10)	0.020 (3)*	
H7B	0.7429 (18)	0.8668 (17)	−0.0069 (11)	0.020 (3)*	
C8	0.91847 (17)	0.70094 (17)	0.03952 (11)	0.0285 (3)	
H8A	0.870 (2)	0.613 (2)	0.0016 (13)	0.042 (4)*	
H8B	0.959 (2)	0.658 (2)	0.1023 (14)	0.042 (4)*	
C9	1.05723 (19)	0.77308 (19)	−0.01183 (12)	0.0327 (3)	
H9A	1.142 (3)	0.698 (2)	−0.0227 (14)	0.045 (3)*	
H9B	1.106 (2)	0.864 (2)	0.0248 (14)	0.045 (3)*	
H9C	1.013 (2)	0.821 (2)	−0.0742 (15)	0.045 (3)*	
C10	0.66429 (16)	0.43700 (14)	0.18745 (8)	0.0186 (3)	
H10A	0.777 (2)	0.4729 (19)	0.1861 (11)	0.024 (3)*	
H10B	0.6141 (19)	0.4995 (19)	0.2383 (11)	0.024 (3)*	

### Atomic displacement parameters (Å^2^)

**Table:**

	*U*^11^	*U*^22^	*U*^33^	*U*^12^	*U*^13^	*U*^23^
O1	0.0380 (5)	0.0142 (4)	0.0190 (4)	−0.0034 (4)	−0.0123 (4)	0.0048 (3)
N1	0.0182 (5)	0.0116 (4)	0.0141 (4)	−0.0019 (4)	−0.0016 (3)	−0.0011 (3)
C1	0.0168 (5)	0.0139 (5)	0.0129 (5)	0.0001 (4)	−0.0010 (4)	0.0010 (4)
C2	0.0160 (5)	0.0127 (5)	0.0134 (5)	−0.0011 (4)	−0.0008 (4)	−0.0015 (4)
C3	0.0190 (5)	0.0117 (5)	0.0144 (5)	−0.0004 (4)	−0.0012 (4)	0.0015 (4)
C4	0.0229 (6)	0.0135 (5)	0.0170 (5)	0.0012 (4)	−0.0019 (4)	−0.0015 (4)
C5	0.0304 (7)	0.0249 (7)	0.0260 (6)	0.0042 (5)	0.0079 (5)	0.0005 (5)
C6	0.0368 (8)	0.0409 (9)	0.0340 (8)	0.0107 (7)	0.0087 (7)	−0.0052 (7)
C7	0.0211 (6)	0.0166 (5)	0.0177 (5)	−0.0042 (4)	−0.0005 (4)	0.0005 (4)
C8	0.0241 (6)	0.0219 (6)	0.0401 (8)	−0.0012 (5)	0.0062 (6)	0.0024 (6)
C9	0.0269 (7)	0.0320 (8)	0.0404 (8)	−0.0047 (6)	0.0098 (6)	−0.0032 (6)
C10	0.0269 (6)	0.0126 (5)	0.0152 (5)	−0.0020 (5)	−0.0062 (4)	0.0016 (4)

### Geometric parameters (Å, º)

**Table:**

O1—C10	1.4214 (14)	C5—H5B	0.977 (18)
O1—H1	0.88 (2)	C6—H6A	0.99 (2)
N1—C2	1.4577 (13)	C6—H6B	0.97 (2)
N1—C7	1.4787 (15)	C6—H6C	1.00 (2)
N1—C4	1.4825 (15)	C7—C8	1.5194 (18)
C1—C3^i^	1.3912 (15)	C7—H7A	1.001 (16)
C1—C2	1.3964 (15)	C7—H7B	0.997 (15)
C1—C10	1.5094 (15)	C8—C9	1.5191 (19)
C2—C3	1.3931 (15)	C8—H8A	0.99 (2)
C3—H3	0.958 (14)	C8—H8B	0.99 (2)
C4—C5	1.5171 (18)	C9—H9A	0.97 (2)
C4—H4A	1.025 (15)	C9—H9B	0.99 (2)
C4—H4B	0.982 (15)	C9—H9C	1.01 (2)
C5—C6	1.524 (2)	C10—H10A	0.980 (16)
C5—H5A	0.970 (19)	C10—H10B	0.999 (16)
			
C10—O1—H1	107.6 (13)	C5—C6—H6C	109.6 (12)
C2—N1—C7	110.57 (9)	H6A—C6—H6C	109.8 (17)
C2—N1—C4	111.02 (9)	H6B—C6—H6C	108.3 (17)
C7—N1—C4	111.04 (9)	N1—C7—C8	112.37 (10)
C3^i^—C1—C2	118.48 (10)	N1—C7—H7A	107.4 (9)
C3^i^—C1—C10	120.84 (10)	C8—C7—H7A	109.2 (9)
C2—C1—C10	120.66 (10)	N1—C7—H7B	110.9 (9)
C3—C2—C1	119.90 (10)	C8—C7—H7B	110.2 (9)
C3—C2—N1	120.22 (10)	H7A—C7—H7B	106.6 (12)
C1—C2—N1	119.88 (9)	C9—C8—C7	112.02 (12)
C1^i^—C3—C2	121.62 (10)	C9—C8—H8A	109.9 (11)
C1^i^—C3—H3	118.9 (8)	C7—C8—H8A	108.2 (11)
C2—C3—H3	119.5 (8)	C9—C8—H8B	110.7 (11)
N1—C4—C5	111.94 (10)	C7—C8—H8B	108.4 (11)
N1—C4—H4A	112.2 (8)	H8A—C8—H8B	107.5 (15)
C5—C4—H4A	109.3 (8)	C8—C9—H9A	112.8 (12)
N1—C4—H4B	106.4 (9)	C8—C9—H9B	111.0 (11)
C5—C4—H4B	110.4 (9)	H9A—C9—H9B	108.4 (16)
H4A—C4—H4B	106.4 (12)	C8—C9—H9C	109.4 (11)
C4—C5—C6	112.13 (12)	H9A—C9—H9C	110.4 (16)
C4—C5—H5A	108.8 (10)	H9B—C9—H9C	104.5 (16)
C6—C5—H5A	109.3 (10)	O1—C10—C1	110.23 (9)
C4—C5—H5B	108.5 (10)	O1—C10—H10A	111.1 (9)
C6—C5—H5B	110.8 (10)	C1—C10—H10A	108.3 (9)
H5A—C5—H5B	107.2 (15)	O1—C10—H10B	110.0 (9)
C5—C6—H6A	110.6 (13)	C1—C10—H10B	110.4 (9)
C5—C6—H6B	111.6 (12)	H10A—C10—H10B	106.9 (13)
H6A—C6—H6B	106.9 (17)		
			
C3^i^—C1—C2—C3	−0.09 (18)	N1—C2—C3—C1^i^	179.23 (10)
C10—C1—C2—C3	178.66 (11)	C2—N1—C4—C5	69.20 (12)
C3^i^—C1—C2—N1	−179.23 (10)	C7—N1—C4—C5	−167.35 (10)
C10—C1—C2—N1	−0.48 (16)	N1—C4—C5—C6	175.43 (12)
C7—N1—C2—C3	−61.82 (13)	C2—N1—C7—C8	−61.45 (13)
C4—N1—C2—C3	61.89 (13)	C4—N1—C7—C8	174.85 (10)
C7—N1—C2—C1	117.32 (11)	N1—C7—C8—C9	−178.53 (11)
C4—N1—C2—C1	−118.97 (11)	C3^i^—C1—C10—O1	0.79 (16)
C1—C2—C3—C1^i^	0.09 (19)	C2—C1—C10—O1	−177.92 (10)

Symmetry code: (i) −*x*+1, −*y*+1, −*z*.

### Hydrogen-bond geometry (Å, º)

**Table:**

*D*—H···*A*	*D*—H	H···*A*	*D*···*A*	*D*—H···*A*
O1—H1···N1^ii^	0.88 (2)	2.05 (2)	2.9269 (13)	171.7 (18)

Symmetry code: (ii) −*x*+3/2, *y*−1/2, −*z*+1/2.
